# The Influence of Polysaccharide Coating on the Physicochemical Parameters and Cytotoxicity of Silica Nanoparticles for Hydrophilic Biomolecules Delivery

**DOI:** 10.3390/nano9081081

**Published:** 2019-07-27

**Authors:** Tatiana Andreani, Joana F. Fangueiro, Patrícia Severino, Ana Luiza R. de Souza, Carlos Martins-Gomes, Paula M. V. Fernandes, Ana C. Calpena, Maria P. Gremião, Eliana B. Souto, Amélia M. Silva

**Affiliations:** 1 CITAB—Centre for Research and Technology of Agro-Environmental and Biological Sciences, University of Trás-os-Montes e Alto Douro, Quinta de Prados, 5001-801 Vila Real, Portugal; 2 Department of Biology and Environment, University of Trás-os-Montes e Alto Douro, 5001-801 Vila Real, Portugal; 3 CIQUP—Research Center in Chemistry, Department of Chemistry and Biochemistry, Faculty of Sciences, Porto University, Rua do Campo Alegre, s/n, 4169-007 Porto, Portugal; 4 Institute of Technology and Research, University of Tiradentes, Avenida Murilo Dantas, Farolândia, 49010-390 Aracaju, Brazil; 5 Faculty of Pharmaceutical Sciences, Universidade Estadual Paulista, UNESP, Rodovia Araraquara-Jau, Km. 01, 14801-902 Araraquara, São Paulo, Brazil; 6 Biopharmacy and Pharmacokinetic Unit, Pharmacy and Pharmaceutical Technology Department, School of Pharmacy, University of Barcelona, Av. Joan XXIII, s/n, 8028 Barcelona, Spain; 7 Department of Pharmaceutical Technology, Faculty of Pharmacy, University of Coimbra (FFUC), Pólo das Ciências da Saúde, Azinhaga de Santa Comba, 3000-548 Coimbra, Portugal; 8 CEB—Centre of Biological Engineering, University of Minho, Campus de Gualtar 4710-057 Braga, Portugal

**Keywords:** silica nanoparticles, polysaccharides, insulin, hydrophilic biomolecules, factorial design, kinetic studies, cell toxicity

## Abstract

The present work reports the effect of polysaccharides (chitosan and sodium alginate) on silica nanoparticles (SiNP) for hydrophilic molecules delivery taking insulin as model drug. The influence of tetraethyl orthosilicate (TEOS) and homogenization speed on SiNP properties was assessed by a 2^2^ factorial design achieving as optimal parameters: 0.43 mol/L of TEOS and homogenization speed of 5000 rpm. SiNP mean particle size (Z-Ave) was of 256.6 nm and polydispersity index (PI) of 0.218. SiNP coated with chitosan (SiNP-CH) or sodium alginate (SiNP-SA) increased insulin association efficacy; reaching 84.6% (SiNP-SA) and 90.8% (SiNP-CH). However, coated SiNP released 50–60% of the peptide during the first 45 min at acidic environment, while uncoated SiNP only released ~30%. Similar results were obtained at pH 6.8. The low Akaike’s (AIC) values indicated that drug release followed Peppas model for SiNP-SA and second order for uncoated SiNP and SiNP-CH (pH 2.0). At pH 6.8, the best fitting was Boltzmann for Ins-SiNP. However, SiNP-CH and SiNP-SA showed a first-order behavior. Cytotoxicity of nanoparticles, assessed in Caco-2 and HepG2 cells, showed that 100 to 500 µg/mL SiNP-CH and SiNP-SA slightly decreased cell viability, comparing with SiNP. In conclusion, coating SiNP with selected polysaccharides influenced the nanoparticles physicochemical properties, the insulin release, and the effect of these nanoparticles on cell viability.

## 1. Introduction

Although the oral ingestion of therapeutic drugs is the preferred route of administration, the development of oral drug delivery systems is encountering serious limitations such as, high molecular weight and poor permeability across the gastrointestinal epithelium resulting in low bioavailability of orally administered drugs [1,2]. Furthermore, the acidic environment in the stomach and the presence of proteolytic enzymes located throughout the gastrointestinal tract (GIT) are also obstacles faced in the oral administration of drugs. The degradation of therapeutic protein molecules can be carried out by the stomach enzymes, such as pepsin and by the intestinal enzymes, such as trypsin, chymotrypsin and carboxypeptidases, and by the environmental pH [3]. To overcome such obstacles and increase the oral bioavailability of the therapeutic molecules, a wide range of strategies using different type of nanoparticles as drug delivery carriers has been adopted as shown in several works [4,5,6,7]. In this way, the use of silica nanoparticles (SiNP) as platform for effective targeting therapy has gained a special attention due to their well-defined properties such as high surface area, high biocompatibility and easy surface functionalization/bioconjugation [8] being an excellent system for the delivery of biomolecules, such as proteins and peptides [9,10,11]. The synthesis of SiNP can be governed by the presence of silanol groups (–Si–OH) and siloxanes onto their surface [12]. The functional –SiOH group can react with several organic compounds, including amine, carboxyl or thiol groups [13]. Some studies have shown that the functionalization of silica surface is the most important strategy to modify the release of biomolecules from SiNP. For example, Song et al. (2007) demonstrated that amino functionalized SiNP adsorb more bovine serum albumin (BSA) in comparison to non-functionalized SiNP due to a strong interaction between the negative charge in proteins and positive charge of the amines [14]. Also, Nairi et al. (2018) showed that mesoporous silica nanoparticles (MSiNP) functionalized with hyaluronic acid and chitosan modulate their interaction with BSA due to the van der Waals forces [15]. Another interesting study showed that MSiNP functionalized with hyaluronic acid can be easily up taken by 3T3 mouse fibroblast cells compared with MSiNP functionalized with chitosan [16]. Therefore, the functionalization can influence the physicochemical properties, pharmacokinetic, cellular uptake and toxicity of SiNP. In this context, several studies have reported the application of SiNP as carriers for the oral delivery of hydrophilic biomolecules. Mohanraj et al., (2010) developed a hybrid nanomaterial based on SiNP coated liposomes for insulin delivery. Coated liposomes showed high stability in simulated lipid digestive media and reduced the insulin release in low pH in comparison to uncoated liposomes [17]. Oroval et al., (2017) reported that β-cyclodextrin-modified enzyme glucose oxidase used as gatekeepers in MSiNP for insulin release showed to be sensitive to the glucose concentration [18]. In the same way, insulin was loaded into MSiNP modified with carboxyphenylboric acid and containing sodium alginate onto their surface as gatekeeper. In the presence of high glucose concentration, the boronate esters dissociate leading to the opening of mesoporous channels with subsequent release of insulin [19]. After developing a glucose-responsive insulin release system based on MSiNP using zinc oxide nanoparticles as capping agent and phenylboronic acid as the glucose response component, Hei et al. (2019) showed the high biocompatibility of these nanoparticles on Chang liver cells at concentrations up to 200 μg/mL. The release of insulin from these nanoparticles was dependent on the monosaccharide concentration [20]. Taking into account the chemical versatility of SiNP associated to their surface modification facilities and their ability to conjugate to hydrophilic molecules, the main aim of the present study was to compare the effect of mucoadhesive compounds, such as polysaccharides (chitosan or sodium alginate) on the properties of SiNP for hydrophilic drug delivery, using insulin as model biomolecule. The design of mucoadhesive nanoparticles to develop oral insulin-delivery systems has received increasing attention in recent years. Mucoadhesive drug-delivery systems are based on the ability of bioadhesion of the certain natural or synthetic material to adhere to mucus membranes [21], targeting a drug to a particular mucus tissue for an extended period of time. Hydrophilic polysaccharides, such as chitosan or sodium alginate, have been widely used to improve the penetration of loaded proteins/peptides in the intestinal mucosa due to the mucoadhesive and absorption-enhancing properties of the former [22].

When developing nanoparticles, in particular for drug delivery and targeting, particle agglomeration should be critically controlled, given the nanoparticles’ sensitivity to the experimental conditions [23]. The design of particles for oral administration requires a certain control of the mean size and size distribution since these parameters may influence the particle uptake from the epithelial cells [24], adhesion [25] and cell transfection [26]. Therefore, in the present study, a 2-level-2 factor factorial design experiment was applied to determine the interaction between the factors on the size and size distribution of SiNP. The influence of polysaccharides on SiNP properties (size, size distribution, surface charge and insulin association efficacy) and on the insulin release profile applying mathematic modelling was investigated. Considering that the major target issues and routes for exposure to oral peptide-loaded nanoparticles are the intestine and the liver cells, we also evaluated the potential health impact of synthesized nanoparticles using human cell lines from intestine (Caco-2) and from liver (HepG2).

## 2. Materials and Methods

### 2.1. Materials

Tetraethyl orthosilicate (TEOS, 98%) and 25% NH_3_ solution were purchased from Merck (Darmstadt, Germany). Low molecular weight chitosan (235 g/mol, deacetylation degree of 78.5%), ethanol 99.9%, trehalose dehydrate and bovine serum albumin (BSA) were purchased from Sigma–Aldrich (Steinhein, Germany). Sodium alginate (198.11 g/mol) was purchased from VWR Portugal (Carnaxide, Portugal). Solution of 100 IU/mL of human insulin (Humulin^®^ R) was purchased from Eli Lilly (Lisbon, Portugal). Dulbecco’s modified Eagle’s medium (DMEM), fetal bovine serum (FBS), penicillin/streptomycin, L-glutamine, 0.05% trypsin–EDTA and Alamar Blue (AB) were purchased from Gibco (Alfagene, Invitrogen, Portugal). HepG2 (Human hepatocellular carcinoma cell line; ATCC^®^ Number: HB-8065TM) was a gift from Professor Carlos Palmeira (CNC-UC, Coimbra, Portugal) and Caco-2 (Human colon adenocarcinoma cell line) was purchased from Cell Lines Service (CLS, Eppelheim, Germany). Ultra-purified water was obtained from MiliQ^®^ Plus system (Millipore, Germany).

### 2.2. Synthesis of and Optimization of Silica Nanoparticles (SiNP) by Factorial Design

SiNP were synthesized at room temperature via hydrolysis and condensation of precursor TEOS under high shear homogenization (HSH) (18G impeller, Ultra-Turrax, IKA, T25). TEOS, ethanol and NH_3_ was mixed for 2 h. The resulting nanoparticles were centrifuged and washed with a mixture of ethanol and ultra-purified water (1:1, V/V) by 2 cycles at 11,773× *g* (Spectrafuge16 M, Lambnet International, Inc.) for 5 min and the pellet was freeze-dried in the presence of trehalose (10%, w/V) to prevent particle aggregation [27].

To maximize the yield of nanoparticles’ production with a minimum of experiments, a factorial design approach was applied. The influence of TEOS and different homogenization speeds on the particles’ properties were evaluated using a 2^2^ factorial design with triplicate of central point, composed of 2 variables which were set at 2-levels each. A total of 7 preliminary experimental combinations were undertaken. For each independent variable, 3 levels were used to describe the interaction between them as summarized in Table 1. The independent variables were the concentration of TEOS and the homogenization speed, while the established dependent variables were Z-Ave and PI. The effect of each parameter on Z-Ave and PI of SiNP was expressed as mean ± standard deviation (SD).

For statistical comparison, Statistica software 7.0 (Statsoft^®^, Tulsa, OK, USA) was used and one-way analysis of variance (ANOVA) test was employed to determine the significance and interactions between factors. A *p*-value < 0.05 was considered statistically significant.

### 2.3. Coating of SiNP with Polysaccharides and Insulin Association

According to the data obtained from the 2^2^ factorial design, the formulation with best values for Z-Ave and PI was used for coating with polysaccharides and insulin association.

For coating SiNP, TEOS, ethanol and NH_3_ were homogenized by HSH at 5000 rpm for 2 h. SiNP were centrifuged and washed with a mixture of ethanol and ultra-purified water (1:1, V/V) by 2 cycles at 12,000 rpm for 5 min. Then a solution of CH (0.3%, m/V) at pH 4.5, or SA (0.3%, m/V) at pH 4.5 was added to 10 mg of SiNP, stirred for 30 min and centrifuged at 5000 rpm and the pellet was freeze-dried in the presence of trehalose (10%, w/V).

Insulin-loaded SiNP were prepared by adding 1.0 mL of human insulin (100 IU/mL, pH 7.2) to 10 mg of SiNP under gently stirring for 30 min in ice bath. The obtained nanoparticles were centrifuged and freeze-dried as described above.

Insulin-loaded SiNP coated with CH or SA were produced by the dissolution of 1.0 mL of human insulin in CH or SA solution, mixed for 30 min under magnetic stirring and then added to 10 mg SiNP under gentle stirring (300 rpm) for more 30 min in ice bath. The final pH of insulin-loaded SiNP coated with polysaccharides was 5.5. The nanoparticles were centrifuged and freeze-dried using trehalose (10%, w/V).

### 2.4. Size, Size Distribution and Zeta Potential Measurements

The mean particle size (Z-Ave) and the polydispersity index (PI) were determined using dynamic light scattering (DLS, Zetasizer Nano NS, Malvern Instruments, Malvern, UK). Each sample was appropriately dissolved with ultra-purified water and the Z-Ave and PI were determined at a fixed angle of 173° and temperature of 25 °C. The zeta potential (ZP) of nanoparticles was measured by Laser Doppler anemometry using the same instrument at the same temperature in purified water adjusting conductivity (50 µS/cm) with 0.9% (m/V) NaCl solution. Each reported value was the average of three measurements (mean ± SD).

### 2.5. Association Efficacy (AE)

The AE was determined in triplicate, using an indirect method after separation by centrifugation of the particles from the aqueous medium containing the non-associated insulin. The amount of insulin associated with nanoparticles was calculated by measuring the difference between the total amount of insulin added to the formulation, and the insulin released in the supernatant. Quantification of insulin was performed according to the Bradford method [28], and using bovine serum albumin (BSA) as standard protein. Absorbance was read using a spectrophotometer (Genesis 10S UV-vis Thermo Scientific), at 595 nm. The AE was determined applying the following equation:

(1) $$\mathrm{AE}\;(\%)\;=\frac{Total\;amount\;of\;insulin-insulin\;in\;supernatant}{Total\;amount\;of\;insulin\;}\;\times \;100$$

### 2.6. Insulin Release and Kinetic Studies

To perform the insulin release profile from different nanoparticles under simulated GI conditions, a comparative study was applied in two different pH values (HCl/KCl buffer at pH 2.0 and phosphate-buffered saline (PBS) at pH 6.8) at 37 °C. The experiment was conducted using a Franz glass diffusion cell and the collected insulin was analyzed by high-performance liquid chromatography (HPLC) as previously described [29]. The analysis was performed in a Waters 1525 pump equipment (Waters, Milford, Massachusetts) with an ultraviolet-visible (UV–vis) 2487 detector (Waters^®^). C18 column (5 µm, 250 mm × 4.6 mm, Phenomenex^®^), with a flow rate of 1.0 mL/min, was used under a mobile phase of acetonitrile–water with 0.1% TFA (40:60, V/V). The injection volume was 100 µL and the absorbance of insulin was recorded at 220 nm.

For the evaluation of insulin release kinetics, the obtained release data were fitted to zero order, first order, second order, hyperbolic and Boltzmann models applying the Akaike’s approach [30]. Selection of the best mathematical modeling profile is based on the lowest value of the Akaike information criteria (AIC). The fittings were performed with resource to GraphPad software.

### 2.7. Cytotoxicity Studies

As described above, HepG2 and Caco-2 cell lines were selected to evaluate the impact of synthesized nanoparticles by constituting the major target sites (colon and liver) to which these nanoparticles will be in contact after being taken orally. Cells were maintained in culture with DMEM, supplemented with 2 mM L-glutamine, antibiotics (100 U/mL of penicillin and 100 µg/mL of streptomycin) and 10% (V/V) of FBS, under standard culture conditions (5% CO_2_ in 95% air, with controlled humidity, at 37 °C). Cells were seeded in 96-well microplates at a cell density of 5 × 10^4^ cells/mL (100 µL per well) and incubated at 37 °C under 5% CO_2_ atmosphere, in fresh cell culture medium. After 24 h of seeding, the medium was replaced with FBS-free medium with lyophilized nanoparticles at concentrations of 50, 100, 200 and 500 µg/mL (control was performed with cells only exposed to FBS-free culture media) and the cells (two sets) were incubated with the nanoparticles during 24 h or 48 h at the same experimental conditions used for the cell culture maintenance. The selection of the concentrations (0–500 μg/mL) for the cytotoxicity studies was based on our previous published studies that indicate low toxicity of these NP at the tested concentrations [31]. We also considered the two time points (24 and 48 h) for cell viability evaluation taking into account that colon transit, the slower of all gastrointestinal transit, is of about 40 h, (in healthy individuals). Therefore, these time points would mimic the longer exposure of cells to these nanoparticles. Not forgetting that during intestinal transit, which is not static, the same intestinal portion is not exposed to the same components during the whole period, however unhealthy individuals may have much longer colon residence time. After the incubation time (24 or 48 h), the incubating media was removed and Alamar Blue (AB) solution (10% (V/V) in FBS-free culture media, 100 µL/well) was added and cells were incubated for more 4 h (controlled humidity, 37 °C and 5% CO_2_). Cell viability was assessed by reading the absorbance at 570 nm (reduced form) and at 620 nm (oxidative form), and by calculating the percentage of AB reduction. Cell viability was expressed as percentage of control, as described elsewhere [31], by the mean ± SD, of 3 independent experiments (each one with quadruplicates).

### 2.8. Statistical Analysis

Data were analyzed by one-way ANOVA with Bonferroni’s multiple comparison as post hoc test to compare the significance between the different groups. A *p*-value less than 0.05 was considered statistically significant.

## 3. Results and Discussion

In recent years, we have evaluated the effect of different mucoadhesive polymers (PEG, chitosan and sodium alginate) on silica nanoparticles properties, toxicity and on biomolecules (insulin) release profile. As demonstrated by our previous works, surface modification of silica nanoparticles by mucoadhesive polymers can lead to the different results regarding to the insulin release and interaction of these NP with mucin, biomembrane models and human cell lines. According to our previous published data, in comparison to PEGylated-SiNP uncoated SiNP reduced the insulin release at gastric and intestinal pH, showed higher protection of insulin from thermal degradation, showed better interaction with biomembrane models, as well as showed low toxicity on HepG2 and Caco-2 cells [29,31]. On the other hand, SiNP coated with CH and SA showed better interaction with mucin molecules in vitro when compared with uncoated and PEGylated-SiNP [32]. Therefore, the novelty of this study lies in the comparison of the different systems, such as SiNP, SiNP-CH and SiNP-SA for biomolecules delivery regarding to insulin (drug model) release behavior, release kinetics and cytotoxicity studies with longer time of exposure.

In the experimental design, TEOS concentration and HSH speed were the selected independent variables to evaluate the interaction between these parameters on the Z-Ave and PI of developed SiNP. The 2^2^ factorial design was presented in form of surface response and Pareto chart to find the optimal manufacturing conditions. The particle size and size distribution of all SiNP formulations predicted by the factorial design are depicted in Table 2.

**Figure 1:**
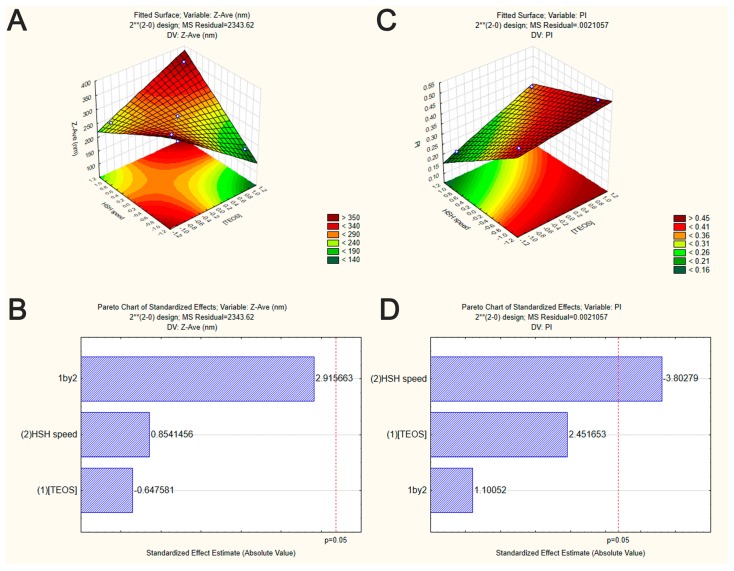
Graphical representation of the interaction between the independent variables on SiNP properties (factorial design experiment set in Table 1). Surface response plots of the effect of TEOS concentration and HSH speed on Z-Ave (**A**) and PI (**C**) of SiNP. Pareto chart of the effect of TEOS concentration and HSH speed on Z-Ave (**B**) and PI (**D**) of SiNP. Vertical lines in Pareto chart indicate the critical effect at 95% confidence level.

From Pareto chart analysis (Figure 1B), no significant effects were observed on the Z-Ave of SiNP when varying TEOS concentration and HSH speed. However, in our study, the interaction among TEOS and HSH demonstrated that SiNP with much smaller sizes were achieved when increasing TEOS concentration and decreasing the HSH speed (Figure 1A).

In the synthesis of SiNP, TEOS monomers are the main source to generate nuclei or primary particles in the aqueous medium. According the classical LaMer’s model to obtain monodispersed particles, nucleation must occur separately from growth [33]. After an initial burst of nucleation, growth occurs through the addition of hydrolyzed monomers to the nuclei surface which occurs throughout primary particle aggregation to form secondary particles (larger particles). In our case, it should be taken in consideration that high TEOS concentration may lead to small particles production due to the presence of a low molar amount of water. Reduced water volume contributes to the decrease of TEOS hydrolysis and condensation which will results in slower growth of hydrolyzed monomers leading to smallest particle sizes [34].

To prepare a variety of nanoparticles with well controlled sizes and shape, the use of stirring methods is almost indispensable. Some studies have shown that stirring may however also favor the aggregation/agglomeration phenomena. For example, Li and Kaner (2006) showed that the mechanical stirring lead to the high aggregation of silica nanoparticles by heterogeneous nucleation at particle surface [35]. However, as indicated by the surface response plots (Figure 1A,C), the produced SiNP showed smaller size when increasing HSH speed. Also, as shown in Pareto chart (Figure 1D and Table 3), only HSH speed had a significant effect on PI values (*p*-value < 0.05). TEOS concentration was no statistically significant, neither the interaction between TEOS and HSH speed (*p*-value ˃ 0.05) (Table 4). Applying high homogenization speeds resulted in small and more monodispersed SiNP as illustrated by Figure 1A,C. Our data are in accordance with those observed by other authors [36]. High homogenization speeds can decrease the particle size due to the increase of energy and the shear stress leading to aggregated particle breakdown [37], maintaining them well dispersed in the medium. Additionally, the high speed intensity of HSH can lead to an increase of local temperature, increasing the nucleation rate and therefore, decreasing, the size of synthesized nanoparticles [23]. These findings drawn from the factorial design showed that the homogenization speed is the most important process for achieving SiNP with narrow size distribution.

**Figure 2:**
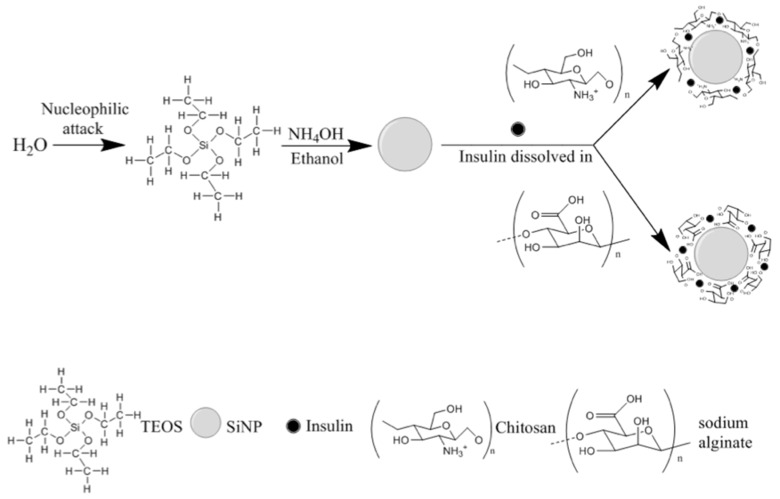
A schematic diagram for the synthesis of hybrid nanoparticles.

From the reported data, an optimal SiNP formulation may be produced with 0.43 mol/L of TEOS and HSH of 5000 rpm. Such conditions generated the synthesis of optimized SiNP with a Z-Ave of 256.6 ± 20.3 nm and a PI of 0.218 ± 0.074. Therefore, F1 was selected as the best formulation to develop hybrid nanoparticles by coating SiNP with chitosan or sodium alginate for insulin association. In this case, the presence of the residual silanol groups in the SiNP surface was used as reactive site to provide efficient surface modification, as well as insulin association (Figure 2).

**Figure 3:**
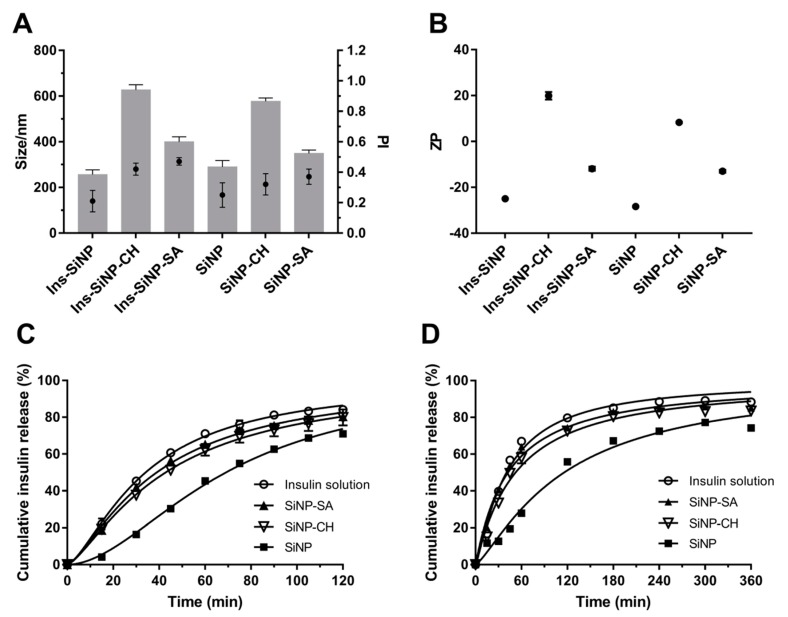
Influence of polysaccharides on nanoparticles properties. Effect of chitosan (CH) and sodium alginate (SA) on Z-Ave (size, nm, denoted as bars) and PI (denoted as full circle) (**A**) and ZP (**B**) of the uncoated and coated formulations. Release profile of free insulin and insulin from nanoparticles in KCl/HCl buffer pH 2.0 (**C**) and PBS pH 6.8 (**D**) at 37 °C. Each point on the curve is the mean of at least three experiments ± SD. Statistical difference between free insulin and insulin loaded nanoparticles is considered with *p* ˂ 0.05. The statistically different formulations at pH 2.0 are: free insulin vs. SiNP in all time points; free insulin vs. SiNP-CH from 15 to 105 min; free insulin vs. SiNP-SA at 60 and 90 min. At pH 6.8, the statistically different formulations are: free insulin vs. SiNP in all time points; free insulin vs. SiNP-CH from 30 to 360 min; free insulin vs. SiNP-SA at 15, 120 and 360 min.

The influence of coating on the properties of SiNP (Z-Ave, PI and ZP) before and after insulin association is shown in Figure 3A,B. Coating SiNP with both polysaccharides led to an increase of Z-Ave and PI. SiNP-CH showed severe particle aggregation exhibiting a Z-Ave around 600 nm and PI around 0.4, while the adsorption of sodium alginate onto SiNP surface led an increment in the particle size of ~390 nm and PI of 0.5 (Figure 3A). This behavior is confirmed by other studies which particle’s agglomeration was observed after coating of nanoparticles with polysaccharides due to the presence of high density and concentration of polymer chains onto nanoparticle surface [38]. As estimated by DLS measurements, compared with coated SiNP, the insulin association led to a slight change on the mean size and size distribution of nanoparticles as depicted in Figure 3A.

The ZP of all nanoparticles is shown in Figure 3B. SiNP exhibited negative charge (−28 mV) due to the presence of deprotonated silanol groups (SiO–) at their surface [39]. However, the ZP of SiNP decreased after coating with sodium alginate (from −28 to −13 mV) and was inverted to positive values after coating with chitosan (from −28 to +8 mV), indicating high evidence of polysaccharides adsorption onto SiNP surface during the processing. It is important to point out that size and surface charge of nanoparticles can influence their intestinal absorption, as well their accumulation in different organs [40]. Consensus among scientific reports has not been reached with regard to the particle size on intestinal uptake. The uptake of larger nanoparticles (˂1000 nm) is generally mediated by M cells of Peyer’s patches [41]. However, some studies report that the oral administration of poly (lactide-co-glycolide) (PLGA) microspheres (1–10 µm) in mice were taken up by Peyer’s patches [42]. By contrast, McClean et al. (1998) using rabbit and rats as animal models showed that only smaller microparticles (˂4 µm) made of poly (lactide) (PLA) were taken up by Peyer’s patches [43].

Apart from particle size, the correlation between the surface charge of nanoparticles and their internalization also still remains questionable. The use of positively charged nanoparticles is usually more efficient to achieve the particle uptake and to escape lysosomal system than negatively or neutrally charged counterparts. Czuba et al. (2018) reported that negatively charged nanoparticles showed better internalization in Caco-2 cells and were more efficient in hyperglycemic rats than nanoparticles with positive charge [44]. Therefore, the different responses regarding to the intestinal absorption are due not only to the intrinsic characteristics of particles, but also to the wide-range of material types, as well as to the type of animals and/or cell models used in the studies of nanoparticle–cell interactions. Although in our study, the size and size distribution of nanoparticles increased after coating, all the particles are still in the nano-range and can be absorbed by intestine cells.

High insulin AE was achieved in all three formulations exhibiting an AE of (71.7 ± 2.4)%, (90.8 ± 5.6)% and (84 ± 5.3)% for Ins-loaded SiNP, Ins-loaded SiNP-CH and Ins-loaded SiNP-SA, respectively. The presence of polysaccharides onto SiNP surface resulted in the increase of insulin AE, in comparison to uncoated nanoparticles. The pH of the dispersion medium modulated the interaction between SiNP, polysaccharide molecules and insulin and consequently the insulin AE. The insulin association to uncoated SiNP was made at pH 7.2. At this pH, the residual silanol groups generate a negatively charged surface which exhibits repulsive forces against insulin (pI = 5.3) [45]. Therefore, the association of insulin with the uncoated SiNP is mediated mainly by physical interactions, such as hydrogen bonds between insulin and silanol groups present onto silica surface. For coated SiNP, the association of insulin was carried out at final pH 5.5 that is closer to the pI of insulin (pI = 5.3) leading to higher AE in comparison to uncoated SiNP. These findings are in agreement with previous data reported by other proteins/peptides showing that proteins have great adsorption ability to polymer surfaces at pH around their pI [46]. This phenomenon can be related to the reduction of electrostatic repulsion and increase of conformation stability of the protein leading to polymer–protein interactions by van der Waal forces or hydrogen bonding [47]. By comparing both coated SiNP, SiNP-CH exhibited higher AE than SiNP-SA. At pH 5.5, chitosan matrix is mainly positively charged due to the protonation state of the amino groups (pKa = 6.3), and thus favors the electrostatic interactions with partially negatively charged insulin. In this case, SiNP also have negative charge and consequently can interact with the positive charges of chitosan leading to high insulin association efficiency. By contrast, sodium alginate presents negative charges at pH 5.5 due to the deprotonation of carboxylic groups (pKa = 3.5), as well as insulin and SiNP resulting in more repulsion between the opposite charges. Therefore, in such experimental conditions, the increase of AE promoted by coating SiNP with alginate could be explained by the non-covalent mechanisms between insulin, polysaccharide and SiNP, such as hydrogen bonding and hydrophobic interactions.

The drug release is another essential parameter for nanoparticle formulations. The insulin release studies from uncoated and coated SiNP were conducted in the gastrointestinal simulated media, such as KCl/HCl buffer (pH 2.0) or PBS (pH 6.8) at 37 °C. The release profiles of insulin from nanoparticles are displayed in Figure 3C,D. It was expected that coating SiNP with polysaccharides, the insulin release would be reduced especially at simulated gastric pH. However, at low pH (2.0), during the initial first 45 min, all formulations released about 50–60% of insulin, except uncoated SiNP, which only 30% was released. As previous stated, silanol groups present onto the SiNP surface are essential sites for insulin association. Such pH 2.0 is near to the pI value of silica, the interaction between insulin and silanol groups can be mediated by hydrogen bonding or hydrophobic interactions. As described above, insulin showed to have more affinity to hydrophilic polysaccharides (as demonstrated by the high AE recorded), resulting in higher insulin adsorption onto coated nanoparticles surface and consequently higher insulin released, in comparison to uncoated SiNP.

The release of macromolecules from alginate matrices under acidic conditions is reduced due to the conversion of sodium alginate into alginic acid [48]. However, in the present study, this conversion could result in the loss of the interaction between the negative charge of alginate and positive charge of insulin, leading to a faster insulin release. When insulin is associated to the SiNP-CH under acidic medium, a fast release was also observed, i.e., 79.9 ± 4.3% within 2 h (please see below). In this case, the chitosan chains become positively charged due to the protonated amino groups. Therefore, there is a weak interaction between insulin and the polysaccharide leading to a faster insulin release. Although chitosan was shown to be an excellent mucoadhesive enhancer, this polysaccharide can dissolve easily at low pH and, consequently, cannot protect insulin from the gastric environment [48]. A study published by Makhlof et al. (2011) described lower insulin release and high peptide stability in acidic medium when loading the peptide in hydroxypropyl methylcellulose phthalate (HPMCP) nanoparticles, in comparison to those made of traditional chitosan nanoparticles [49]. These findings are in agreement with others showing fast release of insulin from chitosan nanoparticles at gastric simulated fluid due to the dissolution of chitosan matrix [50].

Under neutral conditions, 55.8% of insulin was released from uncoated SiNP after 2 h, whereas under simulated gastric conditions the values rose up to 70.9%. This result can be attributed to: (i) the high solubility of insulin at low pH [51], and (ii) to the silica polycondensation reactions that happen at neutral pH, which offer better protein protection and lower silica matrix disintegration [52]. Again, the presence of SA or CH onto SiNP surface contributed to a higher amount of insulin release. At pH 6.8, the insulin release from SiNP-SA could be achieved, due to the swelling of alginate resulting in a soluble salt followed by the matrix disintegration [53]. The same release pattern was observed for SiNP-CH in PBS at pH 6.8. After 60 min, almost 58% of insulin was released from nanoparticles (Figure 5B). This may be due to the negative charges generated in the insulin molecules at neutral pH, as well as due to the deprotonation of amino groups of chitosan chains resulting in reduced electrostatic repulsion and, consequently, in a faster insulin release. In comparison to uncoated SiNP, alginate or chitosan-coated nanoparticles were not able to decrease significantly the release of insulin at low pH, as reported above. Therefore, in this study, not only pH-dependent properties of the polysaccharides can modulate the in vitro release behavior of insulin, but also the interaction between them and the functional groups (Si–OH) present onto SiNP surface.

In order to investigate the mechanism of insulin release from nanoparticles, data obtained from the in vitro release study were fitted to various kinetic equations by analyzing the regression coefficients (r2) and the AIC at pH 2.0 and 6.8 (Table 3). The lower AIC values indicate that at pH 2.0, the release curve for free insulin and for insulin associated to SiNP-SA fit better to the Peppas model (Table 5). On the other hand, the best release mechanism for insulin associated to SiNP (Ins-SiNP) was found to be second order and first order for insulin associated to SiNP-CH. At pH 6.8, the best fitting was Boltzmann for free insulin and for Ins-SiNP, while, Ins-SiNP-CH and Ins-SiNP-SA showed a first-order behavior.

**Figure 4:**
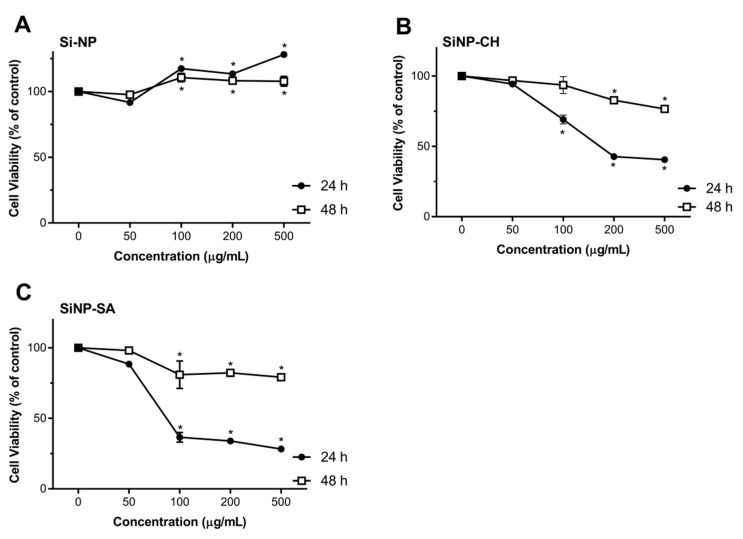
Effect of uncoated and coated SiNP on viability of Caco-2 cells after 24 or 48 h exposure to 50, 100, 200 and 500 µg/mL of SiNP (**A**), SiNP-CH (**B**) and SiNP-CH (**C**). Cell viability is expressed as % of control (n = 8) and data are presented as means ± SD. Statistical difference between control group and formulations is reported as * *p* ˂ 0.05.

Nanoparticles toxicity was evaluated by comparing the proliferation rate and viability of two different cell cultures, HepG2 and Caco-2 cells using the redox indicator Alamar Blue. The results were expressed in cell viability (% of control) after 24 and 48 h of exposure to 50, 100, 200 and 500 µg/mL of nanoparticles dispersion, in FBS-free culture media. After exposing Caco-2 and HepG2 cells to uncoated SiNP, no toxicity response was observed in comparison to the control, in all tested concentrations along the two time points (Figure 4). These results are in agreement with the cytotoxicity studies reported by Tan et al. (2013), in which the hollow SiNP (260 nm) did not induce toxicity to HepG2 cells at concentrations equal or above 400 μg/mL [54]. Zhao et al. (2014) also reported the lack of toxicity to HepG2 cells of hollow SiNP (30 nm) at concentrations up to 200 µg/mL [55]. However, some data reveal severe cytotoxicity and genotoxicity effects of SiNP. For example, Ahmad et al. (2012) described the induction of apoptosis, mediated by the production of reactive oxygen species (ROS) in HepG2 cells when treated with SiNP in the concentration range of 25–200 µg/mL [56]. Recently, Patel et al. (2016) also showed a reduction on Caco-2 cell viability of 66% at 50 µg/mL after 24 h of exposure [57]. The discrepancy of available cytotoxicity data regarding the effects of SiNP on Caco-2 or HepG2 cells can be attributed not only to the properties of SiNP (size, morphology, surface charge, and solubility) but also to the type of SiNP structure (crystalline or amorphous) and their synthesis (fumed or colloidal). Some studies have reported that fumed SiNP synthesized under pyrolysis can generate more SiOH groups onto their surface and hydroxyl radicals (OH–) in comparison to colloidal SiNP synthesized under mild conditions and, therefore, SiOH can interact with biological membranes easily resulting in cell instability and loss of membrane integrity [58]. In our case, the possible explanation for the absence of SiNP toxicity may be due to the low concentration of SiOH groups onto SiNP surface synthesized under room temperature.

**Figure 5:**
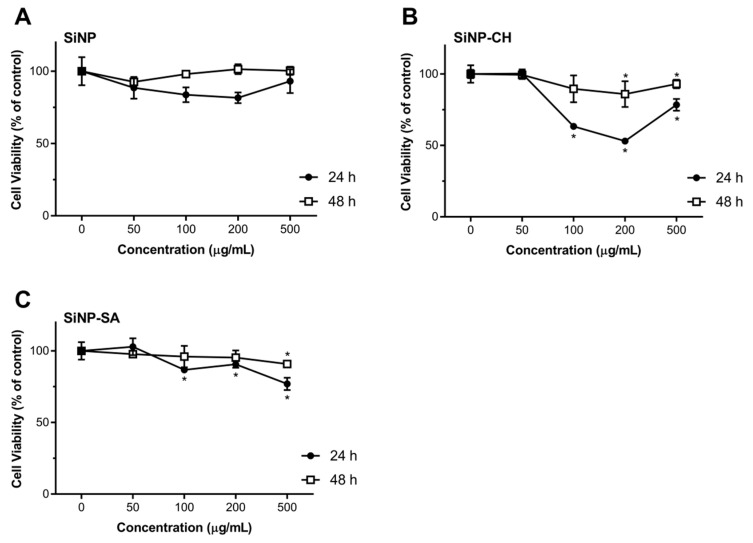
Effect of uncoated and coated SiNP on viability of HepG2 cells after 24 or 48 h exposure to 50, 100, 200 and 500 µg/mL of SiNP (**A**), SiNP-CH (**B**) and SiNP-CH (**C**) Cell viability is expressed as % of control (n = 8) and data are presented as means ± SD. Statistical difference between control group and formulations is reported as * *p* ˂ 0.05.

Exposure of Caco-2 cells to SiNP-CH at concentrations ≥100 µg/mL resulted in a reduction of cell viability after 24 h of exposure (Figure 4). High concentrations reduced the cell viability about 40%. Again, after 48 h, SiNP-CH did not show to be toxic in all tested concentrations. The same results were obtained for HepG2 cells (Figure 5). Concentrations equal or above to 100 µg/mL reduced the cell viability compared to control along after 24 h of exposure, being more pronounced at 200 µg/mL where the cell viability was around 50%, while when the exposure time was 48 h, the viability of HepG2 cells was higher than that obtained in 24 h of exposure. Although the cell viability showed a decrement in both cell lines, the cell viability still higher than 80%, indicating that SiNP-CH are biocompatible. Similar results were obtained by Luo et al. (2017) that showed no toxicity on HepG2 cells of chitosan-coated Fe_3_O_2_ nanoparticles at concentrations up to 200 µg/mL [59] and by Luo et al. (2015) demonstrated lack of toxicity in Caco-2 cells of lipid nanoparticles coated with chitosan [60]. However, Estevez et al. (2014) reported that chitosan-coated selenium nanoparticles decreased the HepG2 cell viability by 40% at concentration of 5 µg/mL [61]. In addition, Vu-Quang et al. (2016) have reported a decrease of cell viability using high chitosan concentrations as coating agent for poly (lactic-co-glycolic acid) nanoparticles [62].

According to the DLS measurements, SiNP-CH showed a positive charge (ZP = +19 mV). Previous studies have demonstrated that cationic compounds lead to cell lysis due to the interaction between the sialic acid residues in the cell membrane and cationic molecules [63]. Therefore, the reduction of cell viability observed after SiNP-CH exposure at high concentrations was attributed to the electrostatic interaction between the negatively charged cell surface and the positively charged amino groups of chitosan, as proposed elsewhere [64].

Concerning to SiNP-SA cytotoxicity, Caco-2 cells were more sensitive than HepG2 cells. As shown in Figure 3, compared to control, the concentrations ≥100 µg/mL reduced significantly the viability of Caco-2 after 24 h (*p* ˂ 0.05). The cytotoxicity of SiNP-SA in Caco-2 cells increased with increasing the treating dose. After 48h of exposure, the cell viability increased around 80% (Figure 4). For HepG2 cells, no toxicity was observed for all concentrations at two time points as shown in Figure 4.

No data were found in the literature about the effect of alginate matrices in Caco-2 and HepG2 cells. However, Wang et al. (2011) observed no cytotoxicity effect on human umbilical cord mesenchymal stem cell of gel containing PLGA nanoparticles coated with alginate at concentrations of 2 mg/mL of PLGA and 30 mg/mL of alginate [65]. Similar results were obtained by Biswas et al. (2015) after exposing HT29 cells to alginate chitosan nanoparticles [66].

## 4. Conclusions

In summary, SiNP synthesis was optimized by 2^2^ factorial design reaching values for Z-Ave of 256.6 ± 20.3 nm and PI of 0.218 ± 0.074 using 0.43 mol/L of TEOS and a homogenization speed of 5000 rpm. SiNP with distinct surface characteristics (chitosan or sodium alginate) were used as carriers for hydrophilic molecules, taking insulin as drug model. Coated SiNP increased the association efficiency of insulin reaching values above 80%. However, the surface modifications of SiNP led to an increase of insulin association efficiency, our studies confirm that coated SiNP were not able to reduce the insulin release at low pH, in comparison to uncoated SiNP. Also, coated SiNP decreased the cell viability mainly in Caco-2 cell lines. The present study reporting the development and characterization of SiNP for hydrophilic biomolecule delivery clearly demonstrated that the physicochemical properties and toxicity of SiNP can be modulated by surface coating with polysaccharides and provides additional insights on the physicochemical properties–release–toxicity relationship, useful for the design of an optimal formulation for oral administration of hydrophilic drugs.

## Figures and Tables

**Table 1:** The values for the lower (−1), central (0) and upper (+1) levels for the variables investigated in the 2^2^ full factorial design for the synthesis of silica nanoparticles (SiNP). (TEOS, tetraethyl orthosilicate; HSH, high shear homogenisation).

Variables	Levels		
	−1	0	+1
TEOS (mol/L)	0.43	0.69	0.95
HSH speed (rpm)	3000	4000	5000

**Table 2:** Experimental results expressed as mean ± standard deviation (SD) for Z-Ave and PI of different SiNP formulations. Influence of TEOS (mol/L) and HSH speed (rpm) on the synthesis of SiNP.

Formulation Code	[TEOS]	HSH Speed	Z-Ave ± SD (nm)	PI ± SD
F1	0.43	5000	256.6 ± 20.3	0.218 ± 0.074
F2	0.43	3000	356.4 ± 112.2	0.443 ± 0.006
F3	0.69	4000	200.7 ± 13.5	0.308 ± 0.021
F4	0.69	4000	292.7 ± 15.2	0.334 ± 0.058
F5	0.69	4000	291.5 ± 35.3	0.349 ± 0.030
F6	0.95	5000	366.4 ± 18.1	0.381 ± 0.005
F7	0.95	3000	183.9 ± 11.1	0.505 ± 0.082

**Table 3:** Evaluation of TEOS concentration ([TEOS]) and HSH speed and their interactions. Analysis of PI by analysis of variance (ANOVA) statistical test.

Factors and Their Interactions	Sum of Squares	Degrees of Freedom	Mean Square	*F-*Value	*p-*Value
(1) [TEOS]	0.012656	1	0.012656	6.01060	0.091545
(2) HSH speed	0.030450	1	0.030450	14.46118	0.031944
(1) by (2)	0.002550	1	0.002550	1.21114	0.351489
Error	0.006317	3	0.002106		
Total	0.051974	6			

**Table 4:** Evaluation of the factors TEOS concentrations ([TEOS]) and HSH speed as well as their interactions. Analysis of the particle size using ANOVA statistical test.

Factors and Their Interactions	Sum of Squares	Degrees of Freedom	Mean Square	*F-*Value	*p-*Value
(1) [TEOS]	982.82	1	982.82	0.419361	0.563396
(2) HSH speed	1709.82	1	1709.82	0.729565	0.455805
(1) by (2)	19923.32	1	19923.32	8.501088	0.061713
Error	7030.86	3	2343.62		
Total	29646.83	6			

**Table 5:** Comparative study of insulin release kinetics from uncoated and coated SiNP at gastric and intestinal conditions.

**Gastric Conditions**				
	**Free Insulin**	**Ins-SiNP**	**Ins-SiNP-CH**	**Ins-SiNP-SA**
**Zero order**				
r^2^	0.9738	0.9929	0.9835	0.9754
AIC	39.86	31.45	35.63	38.98
**First order**				
r^2^	0.9982	0.9944	0.9992	0.9978
AIC	18.29	29.58	11.58	19.63
**Second-order**				
r^2^	0.9985	0.9994	0.9993	0.9982
AIC	19.02	13.01	12.46	20.10
**Boltzmann**				
r^2^	*	0.9999	0.9999	*
AIC	*	14.94	15.04	*
**Hyperbolic**				
r^2^	0.9962	09943	0.9982	0.9961
AIC	81.51	102.75	54.89	81.33
**Peppas**				
r^2^	0.9943	0.9952	0.9965	0.9942
AIC	15.71	15.37	15.08	15.62
**Intestinal Conditions**				
	**Free Insulin**	**Ins-SiNP**	**Ins-SiNP-CH**	**Ins-SiNP-SA**
**Zero order**				
r^2^	0.9427	0.9761	0.9507	0.9481
AIC	61.09	54.21	59.00	58.24
**First order**				
r^2^	0.9986	0.9973	0.9991	0.9993
AIC	29.70	35.06	23.91	21.65
**Second-order**				
r^2^	0.9801	0.9983	0.9861	0.9839
AIC	52.21	29.60	48.35	48.51
**Boltzmann**				
r^2^	0.9992	0.9993	0.9993	*
AIC	28.41	25.75	25.16	*
**Hyperbolic**				
r^2^	0.9937	0.9947	0.9957	0.9965
AIC	39.04	39.17	34.67	31.40
**Peppas**				
r^2^	0.9443	0.9877	0.9794	0.9791
AIC	53.04	47.52	50.28	49.12

* Kinetic fitting model interrupted.
